# Chitosan and Nano-Chitosan for Management of *Harpophora maydis*: Approaches for Investigating Antifungal Activity, Pathogenicity, Maize-Resistant Lines, and Molecular Diagnosis of Plant Infection

**DOI:** 10.3390/jof8050509

**Published:** 2022-05-16

**Authors:** Eman O. Hassan, Tahsin Shoala, Amany M. F. Attia, Omnia A. M. Badr, Sabry Y. M. Mahmoud, Eman S. H. Farrag, Ibrahim A. I. EL-Fiki

**Affiliations:** 1 Department of Plant Pathology, Faculty of Agriculture, Benha University, Banha 13511, Egypt; eman.ali@fagr.bu.edu.eg (E.O.H.); ibrahim.elfiki@fagr.bu.edu.eg (I.A.I.E.-F.); 2 Environmental Biotechnology Department, College of Biotechnology, Misr University for Science and Technology, Giza 12563, Egypt; 3 Department of Plant Pathology, Faculty of Agriculture, Cairo University, Giza 12613, Egypt; amany.farouk@agr.cu.edu.eg; 4 Department of Genetics and Genetic Engineering, Faculty of Agriculture, Benha University, Banha 13511, Egypt; 5 Biology Department, College of Sciences, University of Hafr Al Batin, P.O. Box 1803, Hafar Al Batin 31991, Saudi Arabia; symohamed@uhb.edu.sa; 6 Department of Clinical Laboratory Sciences, College of Applied Medical Science, University of Hafr Al Batin, P.O. Box 1803, Hafr Al Batin 31991, Saudi Arabia; esfarrag@uhb.edu.sa

**Keywords:** *Harpophora maydis*, fungicide, nano-chitosan, disease severity, antifungal activity

## Abstract

The rapid spread of late wilt disease among maize cultivations has resulted in serious economic losses in many countries. *Harpophora maydis* is the main cause of this destructive vascular disease. Here we evaluate the fungicidal activity of chitosan and nano-chitosan against six aggressive isolates of *H. maydis* collected from different Egyptian governorates. Pathogenicity tests for these isolates show that the highest disease severity was found for the Giza isolate. The isolates were tested for their response to the fungicide Permis, chitosan, and nano-chitosan treatments in vitro and in vivo. Nano-chitosan treatments fully inhibited the radial growth of *H. maydis* isolates at concentrations of 5 and 10 mM, compared to the full control growth (9 cm in diameter). On the other hand, in vitro, in vivo, and molecular diagnosis results showed high antifungal activity of chitosan and nano-chitosan compared to the Permis fungicide. Chitosan at the nano and normal scales proved a potent ability to enhance plant resistance in response to *H. maydis*. Disease severity (DS%) was extremely decreased among the tested cultivars by using nano-chitosan; the highest percentage was obtained on Giza 178 cv, where the DS% was 21.7% compared to 42.3% for the control. Meanwhile, the lowest percentage was obtained on Giza 180 cv with DS% 31.2 and the control with 41.3%. The plants treated with nano-chitosan showed the highest growth parameters for all cultivars. Such natural treatments could reduce the impact on the environment as they are non-pollutant natural compounds, protect the plants by reducing fungal activity, and induce plant resistance.

## 1. Introduction

Late wilt, or black bundle disease, is a soil-borne and seed-borne vascular wilt disease of corn that is caused by the fungus *Harpophora maydis* [1]. *H. maydis* is also recognized by the synonyms *Cephalosporium maydis* and *Magnoporthiosis maydis* [2,3]. Late wilt was initially identified as a vascular wilt disease of corn in Egypt in 1960, and it is currently regarded as the most economically damaging fungal disease in commercial maize fields. The disease is gradually spreading and has already been documented by the Centre for Agriculture and Bioscience International (CABI 2020) in countries such as India, Portugal, Spain, Hungary, and it may be present in Kenya. Serious economic losses of up to 40–70 percent occur in infected fields, and these economic losses grow considerably when non-resistant cultivars are used [4,5]. In the absence of tolerant cultivars of corn lines grown, all areas could be seriously impacted by this persistent, soil-borne pathogen because of favorable no-till management and favorable environmental conditions. *H. maydis* can persist on corn stubble for 12–15 months [6,7]. Sclerotia are produced under low humidity and ensure the long-term survival of *H. maydis* (up to 15 months) in no-till residues on the soil surface. Infested seeds can produce plants with late wilt symptoms, infest soil, and then result in the subsequent development of late wilt in healthy seeds grown. In addition, the optimal conditions for corn growth also are optimal for *H. maydis* infection [8,9].

For controlling the late wilt disease of corn, several management strategies have been developed. One of the most effective methods for disease control is using resistant cultivars [3,10]. The National Maize Program at the Agricultural Research Center in Giza, Egypt, has identified many sources of resistance through the screening of thousands of local and exotic germ lines since 1963. The release of such resistant maize varieties resulted in a reduction in late wilt losses in Egypt [11,12]. On the other hand, some races of *H. maydis* appear to be evolving faster than other races, and may respond to the extensive use of resistant varieties of corn in the Nile River Delta, since there is greater variability of *H. maydis* isolates in this intensively cropped area [13]. Moreover, the presence of highly aggressive isolates of *H. maydis* may threaten these resistant maize cultivars. It has also been demonstrated that *H. maydis* could spread in relatively resistant plants with no symptoms and healthy seed performance. The absence of pathogenic symptoms and subsequent protection treatments in such relatively resistant plants may therefore also spread the disease [14].

Chemical fungicides are the most widely used procedure for reducing the pathogen’s impact on commercial production [15,16]. Seed treatments with captan, carbendazim, carboxin and thiram fungicides significantly reduced late wilt disease and increased yields between 11% and 91% in India [17,18]. In contrast, seed treatments consistently failed to control late wilt in Egyptian trials [8]. The failures in Egypt may be due to differences in the chemical sensitivity or virulence of *H. maydis* isolates, chemical formulations evaluated, environmental conditions, or the complexity of the stalk-rot disease complex in Egyptian soils. Systemic fungicides and their fungitoxic products are translocated to corn leaves within 2 days and can persist in corn roots for 90 days; however, field results generally have been disappointing unless the fungicide is applied several times during the growing season [19]. Frequent fungicide application causes hazards to human health and increases environmental pollution. As a result, alternative eco-friendly approaches are needed to control plant diseases [20,21].

Chitosan is an abundant linear biopolymer obtained by alkaline deacetylation of chitin, and it is a homopolymer of β-(1,4)-linked *N*-acetyl-glucosamine units [22]. A few investigators have confirmed that the action of chitosan against pathogenic microorganisms is due to its ability to prevent the growth of pathogens, affecting sporulation, spore germination, and viability. The action of chitosan could be in the form of pathogen cell disturbance or as an inducer for defense responses in the host plant via inducing and inhibiting different biochemical activities during the plant–pathogen interaction [23,24,25,26].

Nanotechnology has enormous potential for improving crop productivity [27], protecting plants [28], and monitoring/detecting plant diseases [29]. Nanoparticles (NPs) have a high surface-to-volume ratio, which boosts their reactivity and potential biochemical activity [30]. Furthermore, nano-based materials are introduced to improve the effectiveness of fungicides and pesticides, allowing for the use of minor dosages. Chitosan nanoparticles have enlarged their functionality in biological activities due to changes in physical and chemical properties such as sizing, surface area, cationic nature, effective operational groups, and greater encapsulation proficiency [31]. Regardless of their potential agricultural implementations, there are few reports on the use of chitosan-NPs in plant disease management, particularly against fungal pathogens.

Despite the enormous economic losses caused by the aggressive isolates of *H. maydis* and the abundance of maize lines in Egyptian sources, research in this field is very limited and restricted to some nations. In this work, we studied the combined use of six genotypes of Egyptian maize lines with chitosan and nano-chitosan as natural fungicides to suppress *H. maydis* infection and increase corn growth and yield. To the best of our knowledge, this is the first study in the Arab Republic of Egypt to combine techniques for evaluating resistant maize lines, antifungal activity, infection, and the molecular response of defense induction.

## 2. Materials and Methods

### 2.1. Pathogen Isolation, Identification and Pathogenicity Test

Corn plants with typical symptoms of the late wilt disease were collected from different areas in Egypt (Nubariya (DMS):30°693776″ N 30°095310″ E; Fayoum (DMS): 29°296281″ N 30°846047″ E; Beheira (DMS):30°678665″ N 30°344802″ E; Minia (DMS): 28°107226″ N 30°740102″ E; Giza (DMS):30°019189″ N 31°205727″ E; and Sharkiya (DMS): 30°724832″ N 31°785659″ E) during the summer of growing season 2018. The pathogen was isolated from the lower internodes of collected symptomatic plants. Stalk cuttings (2 cm length) infected with late wilt lesions were picked and surface-sterilized for 2 min in 1% sodium hypochlorite, removed and rinsed 3 times in sterile distilled water and then dried between sterilized filter papers. Triplicated patch specimens, with 2 mm of each, were plated onto the surface of poured potato dextrose agar (PDA) plates with 0.1% yeast extract according to Zeller [9]. The plates were incubated at 28 ± 2 °C. Five days post-incubation, hyphal tips of the growing colonies were taken randomly and transferred into yeast-PDA plates [4]. The resulting colonies of late wilt fungus were further purified using the hyphal tip technique and sub-cultured several times until the raising of the pure cultures. The isolated fungi were identified based on cultural, microscopic, and morphological characteristics according to Samra et al. [32] and Shoala et al. [33]. The pure pathogenic isolates were recorded and confirmed by the Mycology and Fungi Disease Survey, Plant Pathology Research Institute, ARC, Giza, Egypt. The growing pure cultures were examined microscopically and maintained on yeast-PDA slants amended with yeast extract at 4 °C for further experiments. The pathogenicity test and Koch’s postulates for the isolates were carried out on corn seedlings (Balady var.) in pots (20 cm Ø) using the modified soil inoculation method described by Degani and Cernica [15]. Corn grains were sown in pots (20 cm Ø/2 kg, filled with formalin-sterilized soil/4 grains per pot) and placed in a glasshouse at 28 ± 2 °C with 70% RH. Soil inoculation was done by sowing, where 5 mm Ø agar disks from 5-day old *H. maydis* colonies (grown on yeast-PDA at 28 ± 2 °C as described above) were attached to the upper parts of the roots (2–5 cm beneath the ground surface). The un-inoculated pots served as the control. The planted pots were watered as required. The seedlings were inspected at the full six-leaf stage (30 days post sowing). The severity of the disease progression was evaluated qualitatively using the modified scale of El-Shafey [34]. The re-isolated pure cultures of *H. maydis* were maintained on yeast-PDA slants at 4 °C. The most virulent *H. maydis* isolate was used for the next trials. Pathogenicity tests were carried out under greenhouse conditions at Fac. Agric., Moshtohor, Benha, Egypt.

### 2.2. Plant Materials

Six corn cultivars were used to investigate the host–pathogen interaction between corn plants and the most virulent *H. maydis* isolate. Corn grains of Giza 166, Giza 168, Giza 177, Giza162, Giza 180, and Giza 178 were obtained from the Maize, Sugar and Foliage Crops Res. Dept., Agric. Res. Cent. (ARC), Giza, Egypt. Corn plants were subjected to different treatments of chitosan (C_56_H_103_N_9_O_39_), nano-chitosan and the fungicide Premis Ultra 2.5% (Active ingredient: Triticonazole; chemical name: (5*E*)-5-[(4-chlorophenyl) methylene]-2,2-dimethyl-1-(1*H*-1,2,4-triazol-1-ylmethyl) cyclopentanol).

### 2.3. Synthesis of Chitosan Nanoparticles

Chitosan (CAS number: 9012-76-4) Sigma-Adrich. nanoparticles were prepared via ionotropic chitosan gelation with TPP anions. Chitosan (0.2%) was mixed in ascorbic acid (1%) solution and agitated at room temperature for 1 h (1000 rpm). A TPP stock solution was made by dissolving 0.03 g of TPP in 11 mL of water. Chitosan nanoparticles were formed spontaneously when one ml of TPP stock solution was added dropwise to chitosan solution while stirring (1000 rpm, 1 h) at room temperature. The chitosan nanoparticles were then sonicated for an hour to ensure their tiny size.

### 2.4. Characterization of Nanomaterials

At room temperature, chitosan nanoparticles (CH-NPs) were measured using a dynamic light scattering (DLS) approach using a Zetasizer Nano ZS (Malvern Instruments, UK). An amount of 30 µL of nanoparticles was diluted with 3 mL of water at 25 °C before testing. The mean of the Z-average of three different batches of nanoparticles was used to calculate particle size.

### 2.5. Chitosan Nanoparticles (CH NPs) Transmission Electron Microscopy (TEM) Analysis

For TEM examination, a drop of the solution was deposited on carbon-coated copper grids (CCG) and dried by allowing water to evaporate at room temperature. At the Regional Center for Mycology and Biotechnology (RCMB), Al-Azhar University, electron micrographs were taken using a JEOL GEM-1010 transmission electron microscope at 70 kV [33].

### 2.6. Antifungal Activity of Chitosan Products

#### 2.6.1. In Vitro Tests

The antifungal activity of the prepared chitosan, nano-chitosan and Premis Ultra 2.5% fungicide against the growth of the most virulent *H. maydis* isolate was investigated in vitro. The antifungal activity of tested treatments was tested on PDA plates (90 mm Ø). Chitosan and nano-chitosan were tested aseptically at concentrations of 2.5 mM, 5 mM, and 10 mM by adding them to the melted PDA medium, respectively, to be the final tested concentrations, then poured into Petri dishes in triplicate (3 replicates) for different concentrations and different treatments across the six isolates studied. Then, a disc (3 mm Ø) of the tested *H. maydis* isolate was placed in the center of each plate. PDA plates amended with Premis Ultra 2.5% fungicide (at doses: 1.5, 2.0, and 2.5 µL/mL medium) served as the treated control while a *H. maydis* agar disc only on the center of the plate served as the untreated control. All transferred plates were incubated at 28 ± 2 °C and observed daily until the growth of *H. maydis* covered the whole plate; then, the radial growth (mm) was recorded for each treatment.

#### 2.6.2. In Vivo Tests

The present experiment was carried out on six cultivars of corn plants (Giza 166, Giza 168, Giza 177, Giza 162, Giza 180, and Giza 178) naturally infected with late wilt disease under open field conditions. The relevant area has been known to be infested with late wilt pathogens for many years during the summer growing season 2019/20. In this respect, chitosan treatments that revealed in vitro inhibitory effects on the late wilt pathogen (*H. maydis*) were subjected to in vivo study to confirm their efficacy against late wilt disease and their effect on growth promotion. In this regard, both chitosan and nano-chitosan treatments were applied at concentrations of 5 mM while the fungicide Premis Ultra 2.5% was applied at the recommended dose (2.0 µL/mL). Before planting, grains of targeted corn cultivars were treated individually by soaking for 24 h with chitosan products and fungicide treatments. Grains soaked only in water served as the control treatment. The experimental treatments were laid out in a randomized complete block design (RCBD) with three replicates (plots). Each experimental plot included 3 rows, each 80 cm wide and 6.0 m long. The plot area had a size of about 14.5 m^2^. Each plot contained 75 plants. All agronomic practices endorsed by the Ministry of Agriculture, Egypt, were carried out for the cultivation of corn plants, except fungicide application practices.

### 2.7. Disease Severity Assessments

The disease severity of late wilt was recorded at 90 days post planting, using the modified scale of El-Shafey [34] as follows: 1 = no disease symptoms or slight discoloration in the lower internode; 2 = up to 50% of the lower internode is discolored; 3 = 51–75% of the lower internode is discolored; 4 = 76–100% of the lower internode is discolored; 5 = less than 50% discoloration of the adjacent internode; 6 = more than 50% discoloration of the adjacent internode; 7 = discoloration of three internodes; 8 = discoloration of four internodes; and 9 = discoloration of five or more internodes and premature death of plant. Late wilt disease severity% was assessed according to the following formula:

Disease severity% = Σ(n × v)/9 N) × 100, where: (n) = Number of plants in each category; (v) = Numerical values of symptoms category; (N) = Total number of plants; (9) = Maximum numerical value of symptom category.

### 2.8. Growth Parameters

Vegetative growth parameters, e.g., plant height (cm) and some yield characteristics, e.g., kernel number/plant, row number/ear, kernel number/row, 100-kernel weight (g), and grain yield/plant (g) were recorded after ninety-nine days post-planting in soil infested with *H. maydis.*

### 2.9. Molecular Diagnosis of Late Wilt Pathogenesis

#### 2.9.1. DNA Extraction

Total DNA was extracted from fresh maize leaves of infected and control plants according to the methods of [35,36]. Briefly, samples were grinded with liquid nitrogen and homogenized in a sterilized mortar with 1.5 mL of CTAB extraction buffer, 20 µL of triton x-100, and 100 µL 10% SDS. The sample mixture was transferred to a microcentrifuge tube and incubated at 60 °C for 60 min. Samples were centrifuged at 15,000× *g* rpm for 10 min, and the supernatant was transferred to a new Eppendorf tube. An equal volume of chloroform-isoamyl alcohol was added to the supernatant and then samples were centrifuged at 10,000× *g* rpm for 10 min. The aqueous phase was transferred gently to a new Eppendorf tube. To participate nucleic acids, a 0.45 volume of ice isopropanol was added and mixed thoroughly by inversion and incubated at 25 °C for 1 h. After incubation, samples were centrifuged at 15,000× *g* rpm for 15 min. The supernatant was discarded, the pellet was washed by adding 1 mL of 70% ethanol, and centrifuged at 12,000× *g* rpm for 5 min. The pellet was air-dried for 5 min at room temperature. Then, the elution step was performed using 50 µL of TE buffer. Concentration and purity of purified DNA were measured by BioTek Epoch2 Microplate reader (Thermo Scientific, Waltham, MA, USA). For all samples, DNA purity was >1.8 ± 0.20 under the absorbance ratio A260/A280.

#### 2.9.2. *H. maydis* Detection via qPCR Analysis

Quantitative real-time PCR (qPCR) reactions were performed using Maxima SYBR Green/ROX qPCR master mix (2×) (Thermo Scientific, USA). The reaction was performed in 0.1 mL qPCR strip tubes with optical caps (Gunster Biotech Co., Ltd, Taiwan). Each sample was performed in triplicate including the non-template control to test the presence of primer-dimers. Each reaction consisted of 12.5 μL of SYBR green Master Mix, 11 μL of diluted DNA, and 0.75 μL of each forward and reverse primer pair to reach a final volume of 25 μL. The reaction was completed in AriaMx Real-Time PCR (Agilent Technologies, Santa Clara, CA, USA) using the two-step protocol previously detailed by Abdelatty et al. [37]. For qPCR amplification, the A200a primer pairs were used to amplify a specific *H. maydis* 200 bp piece from an AFLP-derived species-specific fragment (Table 1). To normalize the amount of DNA in eukaryotic mitochondria, the gene coding for the last enzyme in the respiratory electron transport chain—cytochrome c oxidase (COX)—was employed as a “housekeeping” reference gene. The reaction was completed in AriaMx real-time PCR (Agilent Technologies, USA) in triplicate using a two-step protocol: initial denaturation at 95 °C for 10 min, then 40 cycles of denaturation at 95 °C for 15 s followed by annealing/extension at 60 °C for 60 s. A melting curve protocol was run at the end of the PCR by heating at 95 °C for 30 s followed by 65 °C for 30 s and 95 °C for 30 s. Relative gene expression ratios (RQ) between treated and control groups were calculated according to Degani et al. [38] using the formula: RQ = 2^−ΔΔ*C*t^.

### 2.10. Statistical Analysis

All values are presented as a mean of five replicates ± SE. Differences between groups/treatments were evaluated by one-way analysis of variance using SAS software [40]. Differences were considered significant at *p* ≤ 0.05. A Duncan multiple ranges test [41] was used to evaluate the significant differences among means. Furthermore, combined statistical analysis of the two seasons was performed with the least significant difference (L.S.D.) test according to Gomez [42].

## 3. Results

### 3.1. Isolation and Pathogenicity Test of Late Wilt Pathogen

**Figure 1:**
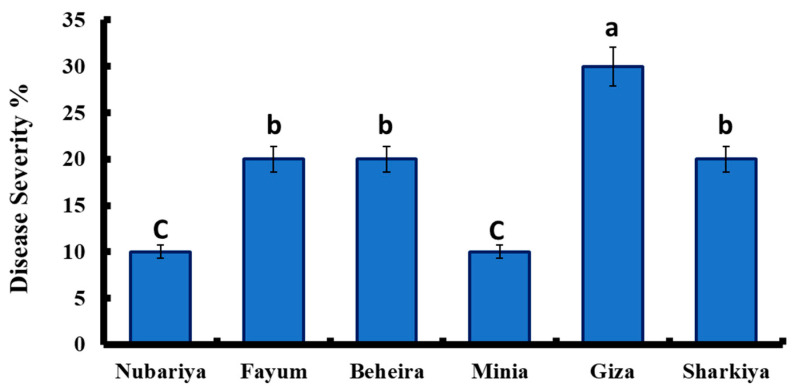
Disease severity percentage of *H. maydis* isolates from different Egyptian governorates. All values are presented as a mean of five replicates + SE. ^a,b,c^ Estimates with the same letters are not significantly different (*p* ˂ 0.05).

As shown in Figure 1, disease severity varied among *H. maydis* isolates based on the source of isolates. The highest disease severity of *H. maydis* isolates was recorded for the isolate from Giza, with 30% severity, while the isolates from Fayum, Beheira, and Sharkiya revealed similar pattern of disease severity (20%). In contrast, the lowest recorded disease severity was found for Nubariya and Minia with 10%.

### 3.2. Characterization of Chitosan Nanoparticles

**Figure 2:**
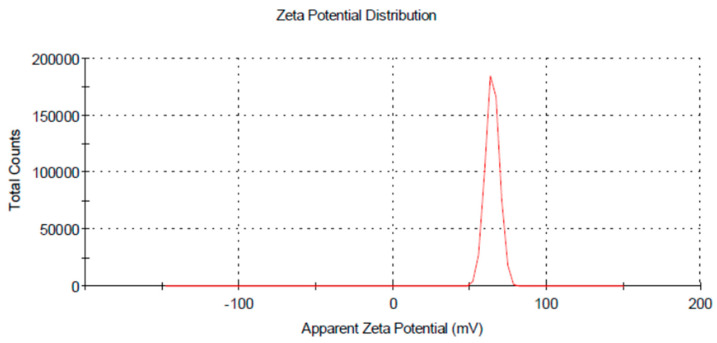
Zeta potential distribution average of chitosan nanoparticles.

**Figure 3:**
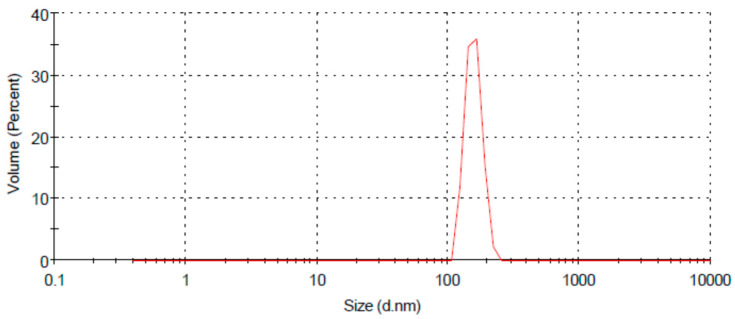
Z average size of chitosan nanoparticles, 100–156 nm.

**Figure 4:**
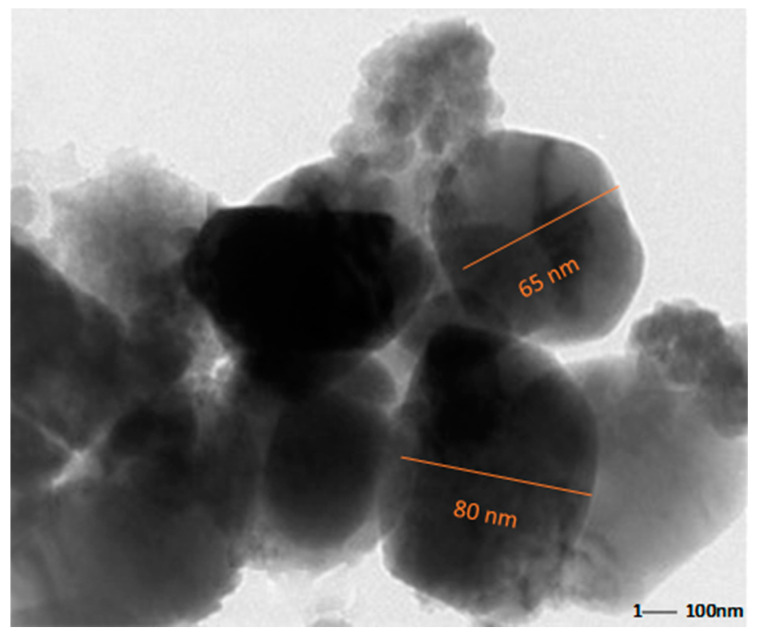
Transmission electron microscopic image of chitosan NPs, ranging from 65–80 nm.

A dynamic light scattering technique was used to understand the size distribution and the stability of prepared chitosan nanoparticles (Figure 2). CH-NPs had a size distribution range mainly within 100–156 nm, as shown in (Figure 3). The Transmission electron microscopic image showed the size of chitosan nanoparticles as ranging from 65–80 nm (Figure 4).

### 3.3. Effect of Fungicide Concentration on In Vitro Antifungal Activity

**Figure 5:**
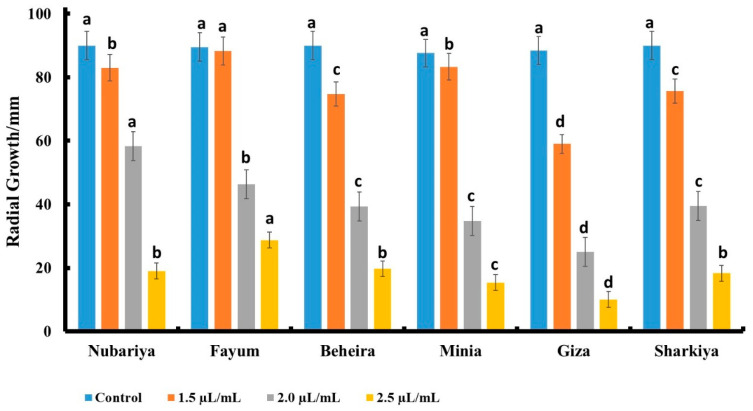
Effect of different concentrations of commercial Premis Ultra 2.5% fungicide on the growth of *H. maydis* isolates from different origin sites at 7 days post-inoculation. All values are presented as a mean of five replicates + SE. ^a,b,c,d^ Estimates with the same letters are not significantly different (*p* ˂ 0.05) among different isolate sources (governorates) for the same concentration.

As shown in Figure 5, the tested fungus isolates were affected by the fungicide Premis Ultra treatments. The highest concentrations of fungicide significantly diminished the radial growth of *H. maydis.*

The isolate from Giza revealed the lowest severity and the best response to the Premis fungicide treatment.

Figure 5 shows that the Giza isolate of *H. maydis* had minimal radial growth of 10 mm in response to 2.5 µL/mL fungicide concentration, while the other concentrations, 2 µL/mL and 1.5 µL/mL, scored 24 mm and 60 mm, respectively, compared to 88 mm in the control. Additionally, the Minia isolate of *H. maydis* showed 15, 35, and 83 mm radial growth in response to 2.5, 2, and 1.5 µL/mL fungicide concentrations, respectively, compared to 88 mm fungal radial growth in the control. Furthermore, Beheira, Sharkiya, and Nubariya isolates showed the same fungal radial growth of 20 mm in response to 2.5 µL/mL fungicide concentration, compared to 90 mm in the control. The Fayum isolate showed 28 mm fungal radial growth in response to 2.5 µL/mL fungicide concentration compared to 90 mm in the control.

**Figure 6:**
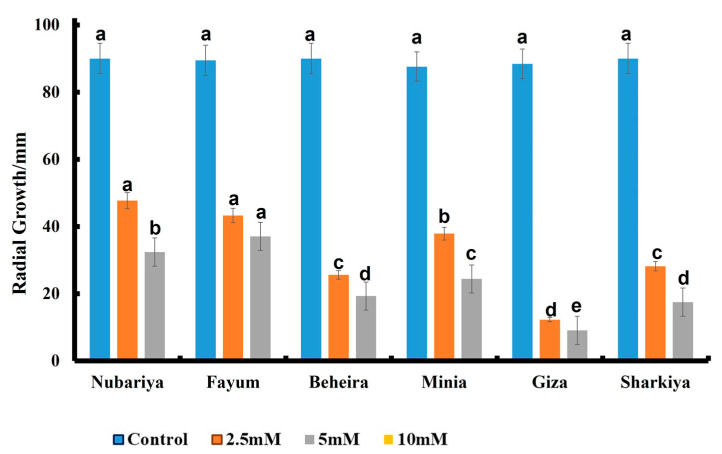
Effect of different concentrations (2.5, 5 and 10 mM) of chitosan on the growth of *H. maydis* isolates from different origin sites at 7 days post-inoculation. All values are presented as a mean of five replicates + SE. ^a,b,c,d^ Estimates with the same letters are not significantly different (*p* ˂ 0.05) among different isolate sources (governorates) for the same concentration.

Figure 6 shows that 10 mM of chitosan suppressed the radial growth of all isolates of the pathogenic fungus H. *maydis*, while 5 mM chitosan significantly suppressed the fungal radial growth to 9, 17, 20, 24, 32, and 36 mm with different isolates from Giza, Beheira, Sharkiya, Minia, Fayum, and Nubariya, respectively, compared to the control. Additionally, the Giza fungal isolate showed 12 mm radial growth in response to 2.5 mM chitosan, compared to 88 mm radial growth in the control. Different pathogenic fungi H. maydis from Beheira, Sharkiya, Minia, Fayum, and Nubariya scored 25 mm, 28 mm, 40 mm, 44 mm and 48 mm, respectively, compared to the control.

**Figure 7:**
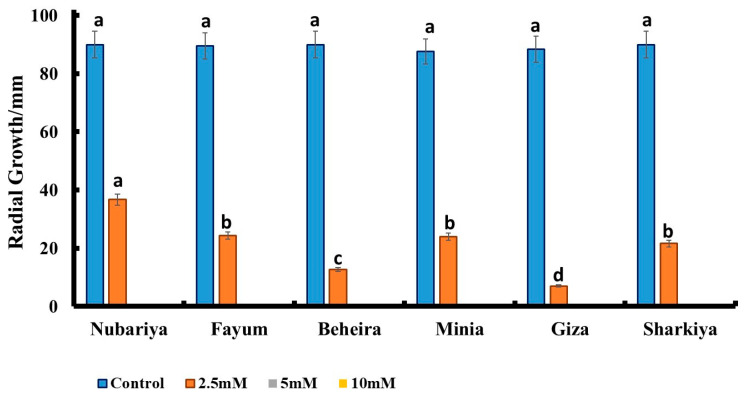
Effect of different concentrations (2.5 mM, 5 mM, 10 mM) of nano-chitosan on the growth of *H. maydis* isolates from different origin sites at 7 days post-inoculation. All values are presented as a mean of five replicates + SE. ^a,b,c,d^ Estimates with the same letters are not significantly different (*p* ˂ 0.05) among different isolate sources (governorates) for the same concentration.

Nano-chitosan treatments inhibited the radial growth of *H. maydis* isolates at concentrations of 5 and 10 mM, while very limited growth was observed at a concentration of 2.5 mM compared to the control group. The *H. maydis* isolate from Giza governate showed the highest response to nano-chitosan treatments, with less severe characteristics, while the *H. maydis* isolate from Nubariya revealed less of a response to nano-chitosan treatments with the highest severity features. The fungal radial growth of the *H. maydis* Giza isolate reached 8 mm in response to 2.5 mM nano-chitosan, while the radial growth of Beheira, Sharkiya, Fayum, Minia, and Nubariya isolates reached 12, 22, 24, 24, and 36 mm, respectively, in response to 2.5 mM nano-chitosan, compared to the control (Figure 7).

### 3.4. In Vivo Antifungal Activity of Chitosan Products

The response of six Egyptian corn cultivars to treatments of chitosan and nano-chitosan at 5 mM concentrations and the Premis Ultra 2.5% fungicide at 2.0 µL/mL (the recommended dose) was evaluated after ninety-nine days of growth under natural infection. As shown in Table 2, all tested treatments revealed a highly significant effect in decreasing the late wilt disease severity (DS%) during the growing season 2019, in comparison with the control group, under open field conditions. In this respect, Giza 178 and Giza 162 cvs recorded the highest decrease in disease severity percentage, respectively, while Giza 177 recorded the lowest decrease in disease severity percentage. Data in the same table reveal that nano-chitosan treatment at the tested concentration had a clear, significant inhibitory effect on disease severity decrements in comparison with the Premis Ultra 2.5% fungicide or control treatments. Interestingly, disease severity was decreased in response to nano-chitosan compared to other treatments and the control. Table 2 shows that the percentage of disease severity in the Giza 178 cultivar reached 21.7%, 29.5%, and 38.6% in the treated seeds with 5 mM chitosan nanoparticles, 5 mM chitosan, and Premis Ultra 2.5%, respectively, compared to 42.3 in the untreated seeds. Additionally, the percentage of disease severity in Giza 162 decreased to 22.2% in response to 5 mM nano-chitosan in comparison with 36.3% in the control. The Giza 166 cultivar showed 22.7%, 31.2%, and 33.3% disease severity in response to chitosan nanoparticles, chitosan, and Premis Ultra 2.5%, respectively, compared to 41.1% for the control. Moreover, treatment with nano-chitosan showed a percentage of 25.5% disease severity in the Giza 177 cultivar, while the disease severity was 34.3% and 38.3% in response to Premis Ultra 2.5% and 5-mM chitosan, respectively, compared to 44.4% in the control. The Giza 180 cultivar showed a disease severity of 27.1%, 31.2%, and 36.3% in response to nano-chitosan, Premis Ultra 2.5%, and chitosan, in that order, compared to 41.3% in the untreated plants. The cultivar Giza 168 scored the highest disease severity with 45.5% in the control, compared to 27.2%, 33.3%, and 44.4% for treatment with nano-chitosan, Premis Ultra 2.5%, and chitosan, respectively. The 100-kernel weight (g) of different cultivars increased in response to nano-chitosan in comparison with chitosan, Premis Ultra 2.5%, and the control.

On the other hand, nano-chitosan showed a significantly convergent result in all yield parameters assessed, compared to the control treatment. In this respect, Giza 162 cv recorded the highest yield parameters, e.g., row no/ear, kernel no/row, 100-kernel weight and grain yield/plant. Meanwhile, Giza 168 and Giza 178 cvs scored the lowest for yield parameters.

**Figure 8:**
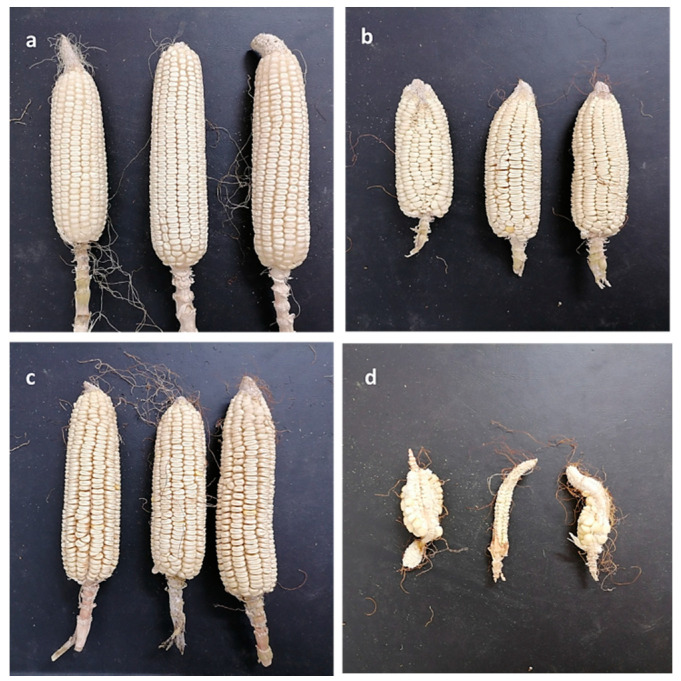
Effect of different treatments, (**a**) nano-chitosan 5 mM, (**b**) chitosan 5 mM, (**c**) fungicide Premis Ultra 2.5%, and (**d**) disease control, on ear size and shape of Giza 166 cv.

Plant heights in all cultivars increased in response to nano-chitosan, except Giza 162, compared to other treatments and control. Interestingly, grain yield per plant reached the highest weights in response to nano-chitosan compared to other treatments and the control. The Giza 162 cultivar showed the highest grain yield weight with 201.2 g in response to nano-chitosan, compared to other cultivars, while the Giza 168 cultivar showed the lowest grain yield weight with 112.1 g in response to nano-chitosan compared to other cultivars. The grain yield increased in all cultivars in response to nano-chitosan, followed by Premis Ultra 2.5%, chitosan, and the control. Data in the same table reveal that nano-chitosan at the tested concentration had clear, significant enhancement effects on the assessed yield parameters in comparison with Premis Ultra 2.5% fungicide treatment or control treatments (Table 2), especially regarding the ear size compared with the other treatments; see Figure 8.

### 3.5. Molecular Diagnosis of Late Wilt Pathogenesis

**Figure 9:**
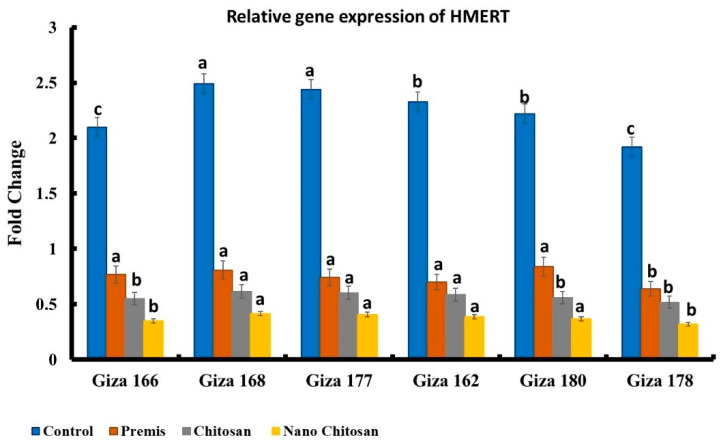
Relative gene expression values of *H. maydis* AFLP-derived species-specific fragment, utilizing A200a primer set against the housekeeping gene of the mitochondria-cytochrome c oxidase, COXI gene. ^a,b,c^ Estimates with the same letters are not significantly different (*p* ˂ 0.05).

The most aggressive isolate of *H. maydis* from Nubariya was subjected to in vivo study against six maize genotypes. Relative gene expression analysis (Figure 9) of these genotypes revealed the successive penetration of *H. maydis* across all targeted genotypes. While Giza 178 exhibited the highest resistant pattern against *H. maydis* infection, the cultivar Giza 168 revealed the highest sensitivity pattern to *H. maydis* infection. Nano-chitosan treatment evidenced higher antifungal activity compared to Premis and control groups.

## 4. Discussion

In this study, isolation trials were carried out on corn plants associated with late wilt disease symptoms from different Egyptian localities (Nubariya, Fayum, Beheira, Minia, Giza, and Sharkiya) and the six resulting *H. maydis* isolates. Pathogenicity tests revealed that tested fungal isolates varied in their virulence. These results could be discussed in the light of findings of Zeller et al. [43], who reported considerable differences in virulence even among the few tested strains of *H. maydis*. We also tested the effect of chitosan and nano-chitosan as fungicides and plant growth promoters against several isolates of *H. maydis,* as well as the effect of the most aggressive isolate against six maize genotypes. In vitro, in vivo, and molecular diagnosis findings confirmed that chitosan and nano-chitosan had the highest antifungal activities at concentrations of 5 mm and 10 mM, respectively, when compared to the Permis fungicide. Chitosan, both in nano and conventional forms, demonstrated a potent ability to boost plant resistance to *H. maydis*. It is widely recognized that in vitro fungicide action is not always a good criterion for in vivo efficacy [44]. Furthermore, it is widely recognized that the use of fungicides is the most effective and traditional way of plant disease control. There are various traditional chemicals that are used to inhibit such pathogenic fungi in this situation. However, numerous phytopathogens have developed resistance to many of the traditional chemicals used to manage them [45,46,47,48]. Consequently, there is an urgent need to produce newer and more effective controlling agents. In this respect, nanotechnology has the potential to deliver more efficient alternatives to conventional fungicides. A number of researchers have validated the effect of chitosan against pathogenic microorganisms due to its capacity to inhibit pathogen growth while also influencing sporulation, germination, and spore viability. Furthermore, chitosan may operate as a cell host disruptor as well as an inducer of defense responses in host plants by stimulating and inhibiting various biochemical activities during the plant–pathogen interaction [23,24,26,49]. Furthermore, the acquired results could be addressed in the context of host–pathogen interaction. Treatment with foliar chitosan and nano-chitosan may stimulate pathways that have a major impact on plant development, physiological activities, secondary metabolites, different routes, and active chemical production. ROS genes are the first defense system route to be activated in response to biotic and abiotic stress, and they can also act as stress protectants, signal translocators, and activators [50].

β-1,3-glucanases are a group of proteolytic enzymes that hydrolyze the 1,3-β-d-glucosidic linkages in β-1,3-glucans (callose) and are identified as one of the popularly known pathogenesis-related (PR) proteins, relating to the PR2 group; they are speedily activated by pathogenic infections as well as salicylic acid (SA), jasmonic acid (JA), ethylene (ET), and chitosan. β-1,3-glucanases are not only generated by pathogen infection, but they also have antifungal action, hydrolyzing fungal β-1,3-glucans, a key cell wall structural element of both fungi and plants [51,52,53]. Chitosan, a natural polymer, has been identified as a powerful biotic adjuvant inducing systemic plant resistance. Chitosan in the form of nanoparticles has not been studied for its phyto-immunogenic action as extensively as chitosan in its natural form. Chitosan nanoparticles (CNP) previously demonstrated substantial antibacterial efficacy against plant pathogens in an in vitro investigation [53].

**Figure 10:**
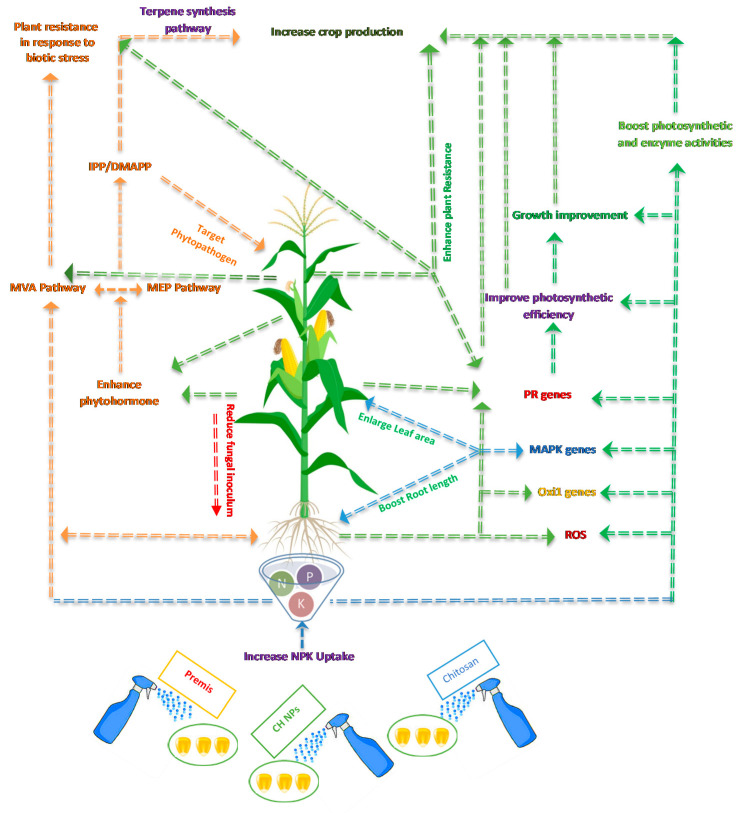
Hypothetical diagram showing different physiological routes in *Zea mays* plants infected with *H. maydis* in response to seed treatments with chitosan, chitosan nanoparticles (CH NPs), and Permis fungicide.

Endogenous treatment of chitosan and chitosan nanoparticles on infected *Zea mays* with *H. maydis* was shown to elicit reactive oxygen species (ROS) and various beneficial plant pathways such as the MAPK cascades pathway [54]. Exogenous administration of chitosan in both its normal and nanoforms was also shown to activate Oxidative signal Inducible1 (Oxi1) (serine/threonine kinase), an AGC family protein kinase. A protein kinase was found that connects ROS accumulation to plant response and resistance to diverse stimuli, as well as oxidative burst-mediated communication in plant roots [55]. On the other hand, MAPK cascades can also improve plant development by enhancing photosynthetic activity via growth hormones [56]. Hence, CNP can operate as an effective plant defense elicitor in in vitro and in vivo contexts and achieve considerably superior immunostimulatory efficiency compared to chitosan (Figure 10).

## 5. Conclusions

Soaking of maize grains with nano-chitosan enhanced plant resistance and crop production in response to the phytopathogenic fungus *H. maydis*. Chitosan nanoparticles reduced disease severity and enhanced all different plant parameters such as plant height and grain yield. Furthermore, Permis fungicide reduced the fungal inoculum, and supported the plants against fungal infection. In vitro results confirmed that chitosan nanoparticles had the highest antifungal activities at concentrations of 5 mm and 10 mM, with zero radial growth for phytopathogen *H. maydis* compared to other treatments. Additionally, in vivo results show that nano-chitosan enhanced crop production, decreased fungal growth, and increased plant resistance compared to other treatments. On the other hand, soaking grains with biodegradable and natural treatments could reduce the impact on the environment and protect the plants by reducing the fungal inoculum and inducing plant resistance. Chitosan in the nano form could be applied as an ecofriendly material to manage specific phytopathogens. Accordingly, we recommend the application of nano-chitosan against the phytopathogenic fungus *H. maydis*. Green technology for a green environment has become an important message for all human beings for protecting the environment.

## Figures and Tables

**Table 1:** Primer pair sequences for qPCR and *H. maydis* detection.

Primer	Sequence	References
AFLP *H. maydis* 200 bp specific fragment (A200a)	5-CCGACGCCTAAAATACAGGA-3	[38]
	5-GGGCTTTTTAGGGCCTTTTT-3	
Cytochrome c oxidase (Cox)	5-GTATGCCACGTCGCATTCCAGA-3	[39]
	5-CAACTACGGATATATAAGRRCCRRAACTG-3	

**Table 2:** Effect of chitosan, nano-chitosan, and Premis Ultra 2.5% fungicide on disease severity and crop parameters of some corn cultivars under natural infection with late wilt disease.

Treatment		DS%	Yield Parameters					
			Plant Height (cm)	Ear No/Plant	Rows No/Ear	Kernels No/Row	100-Kernel Weight (g)	Grain Yield/Plant (g)
Giza 166 cv.	Chitosan 5 mM	31.2	147.7	1.0	12.7	38.0	15.3	73.8
	Nano-chitosan 5 mM	22.7	173.0	1.0	16.0	40.0	23.0	147.2
	Premis Ultra 2.5%	33.3	169.7	1.0	14.0	42.0	19.9	117.0
	Control	41.1	132.5	1.0	12.8	36.5	16.1	75.2
Giza 168 cv.	Chitosan 5 mM	44.4	131.3	1.0	12.3	25.4	13.4	41.9
	Nano-chitosan 5 mM	27.2	166.9	1.0	14.7	38.7	19.7	112.1
	Premis Ultra 2.5%	33.3	145.8	1.0	12.7	30.7	15.1	58.9
	Control	45.4	124.9	1.0	10.7	26.7	14.0	40.0
Giza 177 cv.	Chitosan 5 mM	38.3	142.1	1.0	12.1	34.7	13.7	57.5
	Nano-chitosan 5 mM	25.5	155.9	1.0	14.7	32.0	24.2	113.8
	Premis Ultra 2.5%	34.3	155.6	1.0	12.7	36.0	20.4	93.3
	Control	44.4	142.5	1.0	11.3	33.3	14.7	55.3
Giza 162 cv.	Chitosan 5 mM	33.3	186.7	1.0	13.8	37.3	17.6	90.6
	Nano-chitosan 5 mM	22.2	187.9	1.0	18.0	44.0	25.4	201.2
	Premis Ultra 2.5%	31.2	201.7	1.0	17.3	44.0	21.9	166.7
	Control	36.3	188.0	1.0	14.0	40.0	15.5	86.8
Giza 180 cv.	Chitosan 5 mM	37.6	141.2	1.0	13.2	27.1	10.2	36.5
	Nano-chitosan 5 mM	27.1	166.8	1.0	14.7	34.7	25.6	130.6
	Premis Ultra 2.5%	31.2	153.7	1.0	14.7	30.0	21.7	95.7
	Control	41.3	128.2	1.0	11.3	26.0	11.4	33.5
Giza 178 cv.	Chitosan 5 mM	38.6	97.4	1.0	12.0	20.7	14.2	35.3
	Nano-chitosan 5 mM	21.7	184.6	1.0	15.3	38.0	27.4	159.3
	Premis Ultra 2.5%	29.5	122.3	1.0	14.7	26.7	22.7	89.1
	Control	42.3	101.6	1.0	11.7	18.2	12.4	26.4
L.S.D at 0.05		9.71	6.97	NS	2.11	5.64	6.16	10.22

The tested fungicide was applied at the recommended dose (2.0 µL/mL).

## Data Availability

All data generated and/or analyzed during this study are included in this published article.
